# Supplementary material for: Deletion of wfs1 Impairs Oligodendrocyte Precursor Cells Dorsal Distribution and Myelination Through the wfs1-hmgcs1 Axis in Zebrafish
Source: Biology (Basel). 2026 Aug 16;15(16):1402. doi: 10.3390/biology15161402 (PMC13510151; doi:10.3390/biology15161402)
Supplement: Supplementary file 1 [file biology-15-01402-s001.zip › File S1-Transcriptome sequencing data and differentially expressed genes (DEGs) in wfs1-- zebrafish at 5 dpf/2.KEGG/wfs1KOvsWT/ALL/src/dre00564.html]

dre00564


- dre:560225

- Up regulated genes

560225(2.51169116131343)

- dre:767688

- Down regulated genes

767688(-1.20360432923595)

- dre:406370

- Up regulated genes

406370(7.33922178722892)

- Down regulated genes

406370\_1(-1.88308518916857)
- dre:560649

- Up regulated genes

560649(1.22362655394035)
- dre:767688

- Down regulated genes

767688(-1.20360432923595)
- dre:100148818

- Down regulated genes

100148818(-1.62621488580639)

- dre:724010

- Up regulated genes

724010\_1(10.241655134474)

- Down regulated genes

724010(-6.84228486467569)

- dre:799099

- Up regulated genes

799099\_1(1.05373800076525)

- dre:565743

- Up regulated genes

565743\_1(2.40489825782141)

- dre:767688

- Down regulated genes

767688(-1.20360432923595)

- dre:565743

- Up regulated genes

565743\_1(2.40489825782141)

- dre:406370

- Up regulated genes

406370(7.33922178722892)

- Down regulated genes

406370\_1(-1.88308518916857)
- dre:560649

- Up regulated genes

560649(1.22362655394035)
- dre:767688

- Down regulated genes

767688(-1.20360432923595)
- dre:100148818

- Down regulated genes

100148818(-1.62621488580639)

- dre:572841

- Up regulated genes

572841(1.7118841137558)

- dre:767688

- Down regulated genes

767688(-1.20360432923595)

- dre:560225

- Up regulated genes

560225(2.51169116131343)

- dre:560649

- Up regulated genes

560649(1.22362655394035)

- dre:572841

- Up regulated genes

572841(1.7118841137558)

- dre:560649

- Up regulated genes

560649(1.22362655394035)

- dre:100002015

- Up regulated genes

100002015\_1(2.93882889369414)

- dre:560649

- Up regulated genes

560649(1.22362655394035)

- dre:560649

- Up regulated genes

560649(1.22362655394035)

- dre:572841

- Up regulated genes

572841(1.7118841137558)

- dre:334017

- Up regulated genes

334017\_1(6.06918076131824)

Close
